# Knockin of Cre Gene at Ins2 Locus Reveals No Cre Activity in Mouse Hypothalamic Neurons

**DOI:** 10.1038/srep20438

**Published:** 2016-02-02

**Authors:** Ling Li, Lin Gao, Kejia Wang, Xianhua Ma, Xusheng Chang, Jian-Hui Shi, Ye Zhang, Kai Yin, Zhimin Liu, Yuguang Shi, Zhifang Xie, Weiping J. Zhang

**Affiliations:** 1 Department of Pathophysiology, Second Military Medical University, Shanghai 200433, China; 2 Center for Obesity & Diabetes Research and Innovation, Second Military Medical University, Shanghai 200433, China; 3 Department of General Surgery, Changhai Hospital, Shanghai, China; 4 Department of Endocrinology, Changzheng Hospital, Shanghai, China; 5 Barshop Institute for Longevity and Aging Studies, University of Texas Health Science Center at San Antonio, San Antonio, TX 78245, USA

## Abstract

The recombination efficiency and cell specificity of Cre driver lines are critical for exploring pancreatic β cell biology with the Cre/LoxP approach. Some commonly used Cre lines are based on the short *Ins2* promoter fragment and show recombination activity in hypothalamic neurons; however, whether this stems from endogenous *Ins2* promoter activity remains controversial. In this study, we generated Ins2-Cre knockin mice with a targeted insertion of IRES-Cre at the *Ins2* locus and demonstrated with a cell lineage tracing study that the *Ins2* gene is not transcriptionally active in the hypothalamus. The Ins2-Cre driver line displayed robust Cre expression and activity in pancreatic β cells without significant alterations in insulin expression. In the brain, Cre activity was mainly restricted to the choroid plexus, without significant recombination detected in the hippocampus or hypothalamus by the LacZ or fluorescent tdTomato reporters. Furthermore, Ins2-Cre mice exhibited normal glucose tolerance and insulin secretion upon glucose stimulation *in vivo*. In conclusion, this Ins2-Cre driver line allowed high-fidelity detection of endogenous *Ins2* promoter activity *in vivo*, and the negative activity in the hypothalamus demonstrated that this system is a promising alternative tool for studying β cell biology.

Insulin, the key hormone for glucose and lipid homeostasis, is produced and secreted in a highly regulated manner by β cells in the pancreatic islets of Langerhans. This protein is initially synthesized as a precursor, preproinsulin, which is highly conserved among different species. Unlike most mammals, mice and rats have two non-allelic insulin genes, *Ins1* and *Ins2*, which encode proinsulin I and II, respectively. These two genes have different genomic structures, but both are simultaneously expressed in all β cells at a ratio of 1:2 at the mRNA and protein levels^1^. In mice, these two proteins have two different amino acids in the B chain and three different amino acids in the C chain. The *Ins2* gene is an ortholog to the unique insulin gene present in humans. Targeted disruption of *Ins1* or *Ins2* alone in mice does not lead to developmental or metabolic abnormalities, probably because of a compensatory transcript increase of the intact insulin gene^2^,^3^. However, deletion of both insulin genes causes death from neonatal diabetes in mice^2^.

The two insulin genes have distinct expression patterns in the brain. Most studies have shown that the *Ins2* gene, but not the *Ins1* gene, is expressed in the rodent brain^1^,^4^,^5^; however, the specific cells and expression sites remain controversial. Some investigations have used immunohistochemical staining and *in situ* hybridization to reveal insulin expression in a subset of neurons from mouse, rat and rabbit brains, including the hippocampus and hypothalamus^5^,^6^,^7^,^8^. However, knockin of the LacZ gene at the *Ins2* locus has identified the choroid plexus (CP) as the major site expressing the *Ins2* gene in the brains of newborn mice^9^. This discrepancy is further complicated by observations that the rat *Ins2* promoter (RIP)-driven Cre is active in hypothalamic neurons as well as β cells^10^,^11^,^12^,^13^,^14^,^15^. Furthermore, lineage tracing studies have demonstrated that different RIP-Cre mouse lines have widespread Cre activity in the brain, including the hypothalamus^16^,^17^. Because only the short fragments (~670 bp) of the rat *Ins2* promoter have been used in the RIP-driven Cre lines^18^,^19^,^20^, the extent to which their Cre activity reflects that of endogenous *Ins2* promoter activity remains unclear. However, hypothalamic activity largely limits the application of these Cre driver lines in conditional gene targeting to explore β cell biology, because the hypothalamus is also critically involved in glucose and energy homeostasis^17^,^21^.

To address these two questions, we generated Ins2-Cre knockin mice by targeting *Cre* at the *Ins2* locus. We also used a lineage tracing study based on two distinct reporters to demonstrate that the endogenous *Ins2* promoter is not active in the mouse hypothalamus. Therefore, the generated Ins2-Cre line can be used to study β cell biology.

## Results

### Generation of Ins2-Cre knockin mice

**Figure 1:**
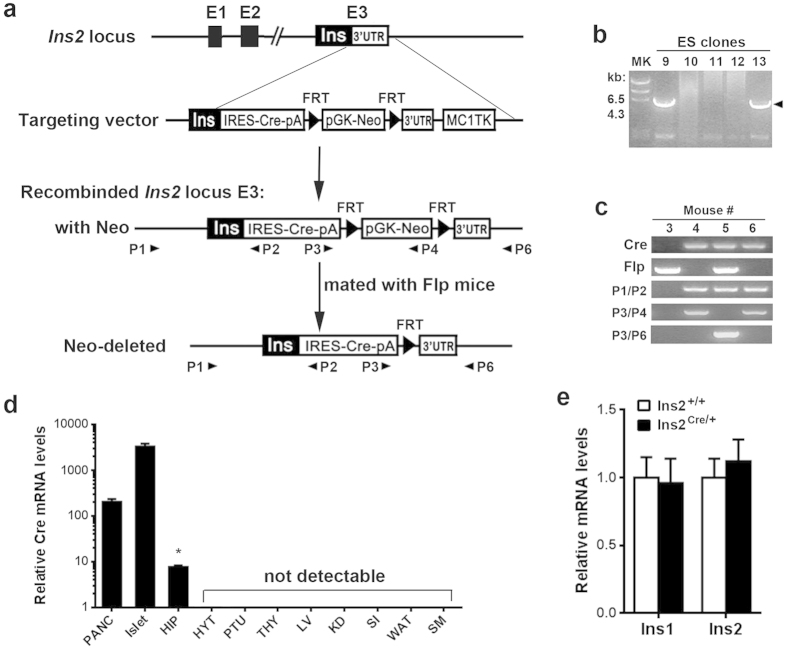
Generation of Ins2-Cre knockin mice. (**a**) Schematic demonstration for knockin of Cre gene at the *Ins2* locus by homologous recombination. The targeting vector contains the IRES-Cre-pA and FRT-flanked Neo cassette, which was inserted between the stop codon and 3′ UTR of the 3^rd^ exon of *Ins2*. After successful homologous recombination in ES cells, the recombined allele continued into the germline. The Neo cassette was removed by crossing onto Flp mice. The genotyping PCR primers (P1-P6) were indicated as their corresponding location of the genome. (**b**) PCR analysis of genomic DNA for the indicated ES clones. A specific band size of 5.6 kb was amplified from positive clones (9 and 13) by PCR using primers P1/P2, indicating homologous recombination at the *Ins2* locus. (**c**) PCR genotyping revealed the identification of the mouse line with the Neo cassette deleted by Flp. In mouse #5, Neo cassette deletion was evidenced by the null PCR amplification using primers P3/P4. (**d**) *Cre* mRNA expression in different tissues from Ins2-Cre mice. PANC, pancreas; HIP, hippocampus; HYT, hypothalamus; LV, liver; KD, kidney; SM, skeletal muscle; WAT, white adipose tissue; PTU, pituitary; THY, thymus; SI, small intestine. *P < 0.001 vs pancreas. N = 4. (**e**) *Ins1* and *Ins2* mRNA expression levels in the islets were not significantly different between control and Ins2-Cre mice. *P* > 0.05 vs control. N = 4.

**Figure 2:**
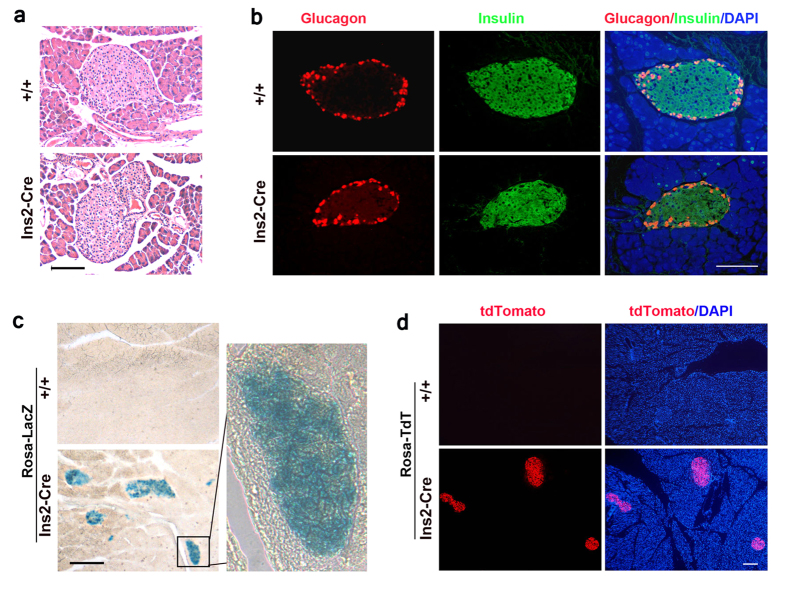
Cre recombination activity in the pancreatic islets of Ins2-Cre mice. (**a–b**) Normal islet architecture of heterozygous Ins2-Cre mice demonstrated by HE staining (**a**) and double immunostaining for glucagon and insulin (**b**). DAPI was used for nuclear counterstaining. Scale bar, 100 μm. (**c**) Cre recombination activity in islets shown by X-gal staining in Ins2-Cre mice crossed onto Rosa-LacZ reporter line. Scale bar, 250 μm. (**d**) Cre recombination activity in islets shown by tdTomato fluorescence in Ins2-Cre mice crossed onto Rosa-tdTomato reporter line (Ai14). TdT, tdTomato. Scale bar, 200 μm.

The Ins2-Cre knockin mice were generating by taking advantage of the internal ribosome entry site (IRES)^22^ to bicistronically express *Cre* together with *Ins2* under the control of the endogenous *Ins2* promoter. To this end, we first constructed a targeting vector by inserting the IRES-Cre sequence with an exogenous bovine growth hormone polyadenylation site (bpA)^23^ downstream the stop codon of the *Ins2* gene (Fig. 1a). After successful homologous recombination in ES cells (Fig. 1b), the targeting vector created the Ins2-Cre allele, which was successfully transmitted to the germline. The resultant offspring were bred with Flp-e mice^24^, thereby removing the pGK-Neo selection cassette downstream the 3′ untranslated region (UTR) of *Ins2* (Fig. 1c). Ins2-Cre mice were born normally and survived into adulthood without gross abnormalities. Characterization of Cre expression in different tissues by quantitative RT-PCR revealed robust expression of *Cre* mRNA in the pancreas and isolated islets from adult Ins2-Cre mice and to a lesser extent in the hippocampus, whereas it was not detected in the hypothalamus, liver, skeletal muscle, white adipose tissue (WAT), kidney, pituitary, thymus, or small intestine (Fig. 1d). Previous reports have identified *Ins2* expression in the hypothalamus and thymus^5^,^25^; therefore, insulin gene expression in these two tissues of Ins2-Cre mice was also measured. mRNA levels of the *Ins1* and *Ins2* genes were scarcely detected in the hypothalamus (Supplementary Figure S1). In contrast, these two genes were expressed in the thymus, although their mRNA levels were extremely low compared with the pancreas and even the hippocampus regarding *Ins2*. However, islet mRNA expression levels of *Ins2* or *Ins1* were not significantly affected by the targeted insertion of IRES-Cre at the *Ins2* locus (Fig. 1e). Hematoxylin-eosin (HE) staining and immunohistochemical staining of insulin and glucagon showed that the Ins2-Cre mice had normal islet architecture (Fig. 2a,b). These data suggested that the *Cre* gene is expressed in Ins2-Cre mice in a tissue-specific manner without significantly compromising insulin gene expression in the islets.

### Characterization of Cre activity

**Figure 3:**
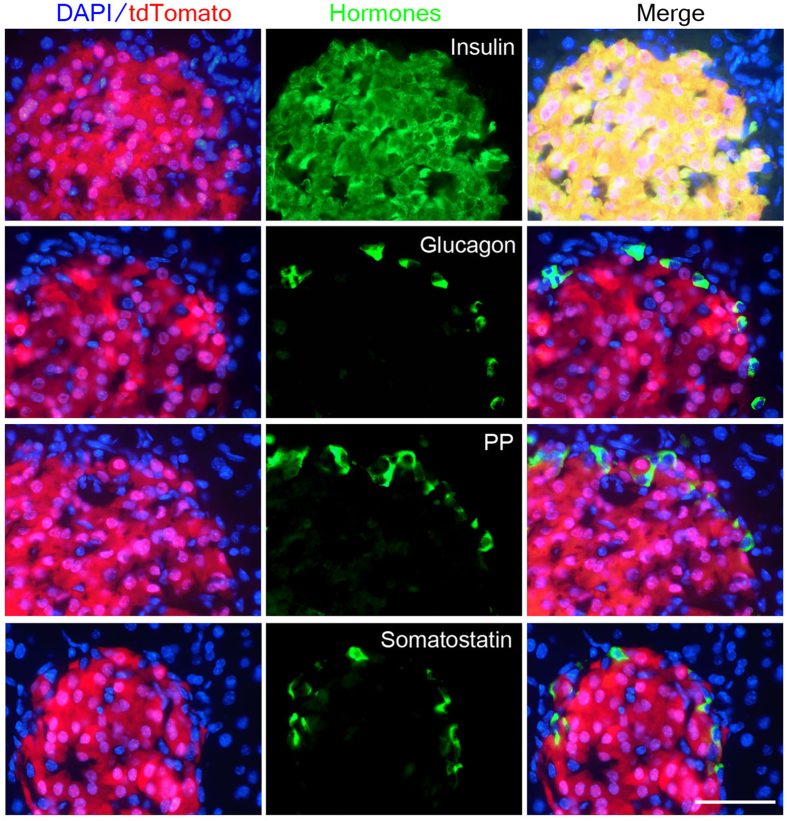
Efficient and specific Cre-mediated recombination in pancreatic β cells from the islets of Ins2-Cre mice. Pancreatic cryosections from Ins2-Cre;Rosa-tdTomato mice were subjected to immunostaining for insulin, glucagon, pancreatic polypeptide (PP), and somatostatin. Cre-mediated recombination occurred only in insulin-expressing cells with high efficiency but not in other hormone-expressing islet cells. Scale bar, 50 μm.

To evaluate the Cre recombination activity, we performed a cell lineage tracing analysis. We first crossed Ins2-Cre mice to the reporter line Rosa26-LacZ, which blocks the production of *LacZ* mRNA by a strong polyadenylation signal flanked by LoxP sites^26^. After Cre-mediated recombination to excise the blocking cassette, the *LacZ* mRNA is synthesized by the ubiquitously active ROSA26 promoter, and as a result, the recombinant β-galactosidase protein can be detected. X-gal staining revealed that Ins2-Cre mice displayed intense blue signals in the islet (Fig. 2c), whereas no signal was detected outside islets in the pancreas. To characterize the Cre activity more intensively in the pancreas, we also took advantage of the Rosa26-tdTomato reporter line Ai14^27^, which expresses the fluorescent protein tdTomato once the stop signal is excised by Cre recombination. Currently, tdTomato is one of the brightest fluorescent proteins available^28^. After crossing Ins2-Cre mice to the Ai14 reporter line, we observed that the bright red fluorescence was consistently and specifically present in the islets but was undetectable outside the islets in the pancreas (Fig. 2d). To further assess the recombination efficiency and specificity in the islets, we performed immunostaining for individual islet hormones on the pancreatic sections from Ins2-Cre/+; Rosa26-tdTomato mice. Approximately 99.8 ± 1.2% of the insulin-expressing cells showed a positive signal of tdTomato fluorescence, whereas no tdTomato signal was detected in islet cell types expressing glucagon, somatostatin, or the pancreatic polypeptide (PP) (Fig. 3). These data demonstrated that Ins2-Cre mediates β cell-specific gene recombination in the pancreas.

**Figure 4:**
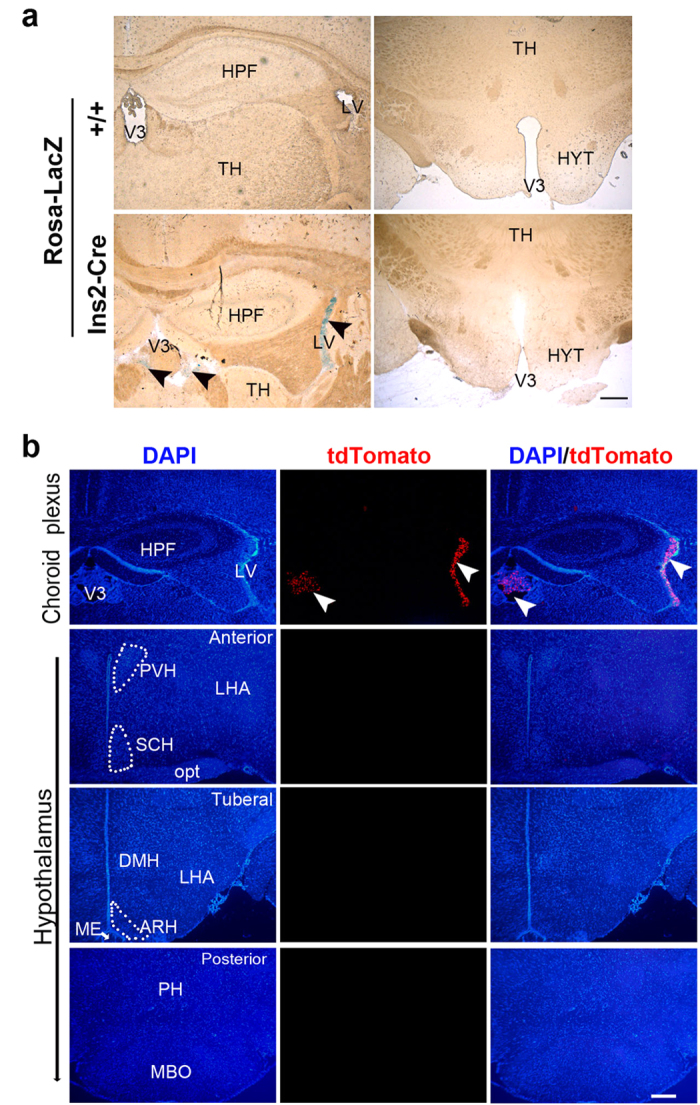
Cre recombination activity detected in the choroid plexus but not in the hypothalamus of Ins2-Cre mice. (**a**) X-gal staining of coronal brain sections showing Cre recombination activity in the choroid plexus of Ins2-Cre;Rosa-LacZ mice. The blue signals in the choroid plexus in the lateral (LV) and third ventricles (V3) are indicated by black arrow heads. No significant staining was detected in the hypothalamus (HYT). Scale bar, 250 μm. (**b**) Representative coronal brain sections of Ins2-Cre;Rosa-tdTomato mice showing that tdTomato was expressed in the choroid plexus in the LV and V3 and undetectable in the anterior, tuberal, and posterior hypothalamus. ARH, arcuate hypothalamic nucleus; DMH, dorsomedial nucleus of the hypothalamus; HPF, hippocampal formation; PVH, paraventricular hypothalamic nucleus; SCH, suprachiasmatic nucleus; LHA, lateral hypothalamic area; MBO, mammillary body; ME, median eminence; Opt, optic tract; PH, posterior hypothalamic nucleus; TH, thalamus. Scale bar, 200 μm.

We then characterized Cre activity in extrapancreatic tissues. With either the LacZ or tdTomato reporter lines, no Cre activity was detected in the liver, skeletal muscle, pituitary, or even thymus of Ins2-Cre mice (Supplementary Fig. S2 and data not shown). As expected, the X-gal staining of brain sections revealed specific blue staining in the CP from Ins2-Cre/+; Rosa26-LacZ mice, but surprisingly not in the hippocampus or hypothalamus (Fig. 4a). Similarly, the Ins2-Cre/+; Rosa26-tdTomato mice displayed bright tdTomato fluorescence in the CP in the lateral, third and fourth ventricles. After careful inspection of consecutive brain sections, we were unable to find any neurons with detectable red fluorescence in the hippocampus, hypothalamus, or other brain regions (Fig. 4b and Supplementary Fig. S3), implying that the *Cre* gene is not active in cells other than the CP in the Ins2-Cre brain. Therefore, it seems that the *Cre* mRNA levels detected in the adult hippocampus by RT-PCR may derive from CP tissues rather than neurons. Collectively, these results suggested that the Ins2-Cre mice mediate tissue-specific Cre recombination in the islet β cells and brain CP.

### Phenotypic analysis of transgenic mice

**Figure 5:**
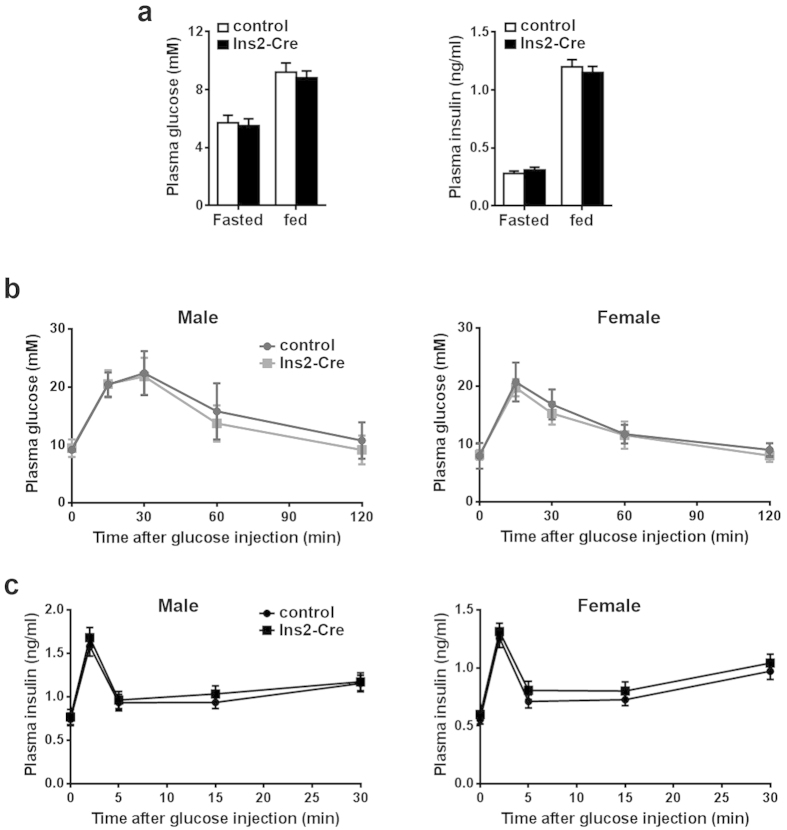
Normal insulin secretion and glucose homeostasis of Ins2-Cre mice. At the age of 3 ~ 4 months old, adult heterozygous male or female Ins2-Cre mice and their age and sex-matched control counterparts were metabolically analyzed. (**a**) Plasma glucose and insulin levels for fasted or fed states in male mice. There was no significant difference between control and Ins2-Cre mice. Each group consisted of four animals. (**b**) Glucose tolerance test on male or female mice. Glucose (2 g/kg body weight) was i.p. injected into the mice, and plasma glucose levels were measured at indicated time points. Each group consisted of six animals. (**c**) Glucose-stimulated insulin secretion after intraperitoneal injection of glucose at indicated time. Glucose (2 g/kg body weight) was i.p. injected into the mice, and plasma glucose levels were measured at indicated time points. Each group consisted of four animals.

To evaluate whether the Ins2-Cre line can be used to functionally study β cell biology, we characterized its metabolic phenotypes. Adult Ins2-Cre mice had normal body weights and plasma glucose and insulin levels under fasted or fed conditions, compared with their wild-type littermate controls (Fig. 5a). The glucose tolerance test (GTT) showed that both male and female Ins2-Cre mice exhibited normal glucose disposal compared with controls (Fig. 5b). Furthermore, the glucose-stimulated insulin secretion (GSIS) test showed that both male and female Ins2-Cre mice displayed a robust insulin secretion response upon glucose stimulation *in vivo*, which was not significantly different from the control groups (Fig. 5c). These data indicated that the Ins2-Cre line has a normal metabolic phenotype.

In summary, we generated Ins2-Cre knockin mice and demonstrated their specific Cre activity in the islet and CP but not the hypothalamus. Our findings suggest that the *Ins2* gene may be not transcriptionally active in the mouse hypothalamus and the Ins2-Cre line may be used to study β cell biology.

## Discussion

Our Cre-mediated lineage tracing study supports the notion that CP is the major extrapancreatic site expressing the *Ins2* gene in mice. The knockin of IRES-Cre is a reliable and powerful approach for lineage tracing studies^22^,^29^. After Cre-mediated LoxP recombination in certain cell types, the expression of the reporter gene is stably inherited by all cell progeny regardless of their differentiation fate. In Ins2-Cre mice, IRES allows bicistronic expression of *Ins2* and *Cre*, therefore Cre expression and activity can consistently reflect the transcriptional activity of the endogenous *Ins2* promoter without potentially sacrificing *Ins2* expression. Using the lineage tracing study based on two distinct reporters (Rosa-LacZ and Ai14), we clearly demonstrated that the *Ins2* gene is transcriptionally active in the CP as well as islets but not in the hippocampus or hypothalamus. Notably, the bright native fluorescence of tdTomato is more sensitive than X-gal staining, and more importantly, it allows direct visualization of fine dendritic structures and axonal projections of the labeled neurons^27^, thus overcoming the potential shortcomings of X-gal staining in detecting some small isolated or diffuse neurons in the brain. The experiments with the tdTomato reporter line did not show obvious red fluorescence in the consecutive hypothalamic sections from Ins2-Cre mice. Therefore, these data strongly suggest that the *Ins2* gene is not expressed in hypothalamic neurons during their development and differentiation. This conclusion is consistent with results from an earlier report based on the knockin of the LacZ gene directly at the *Ins2* locus^9^. However, in RIP-Cre mice, the *Cre* gene is driven by a relatively short fragment of the rat Ins2 promoter (~670 bp)^18^,^19^,^20^. Therefore, it is possible that their Cre activity in the brain, including the hypothalamus, most likely reflects an ectopic leaky expression from a lack of tight transcriptional control.

Insulin action in the brain plays an important role in regulating brain function and systemic metabolism^30^,^31^. However, the relevant source of insulin for the brain is unclear. Targeted disruption of *Ins1* or *Ins2* alone in the mouse does not lead to developmental or metabolic abnormalities^2^, probably due to a compensatory transcript increase of the insulin gene which remained intact, as observed in the pancreas^3^. However, compound deletion of the two insulin genes or destruction of insulin-expressing in cells leads to insulin deficiency, embryonic growth retardation, and newborn lethality^2^,^9^. The expression *Ins2* in the CP raises the possibility that insulin from the CP may have a role in the brain. Interestingly, the CP selectively expresses *Ins2* rather than *Ins1*^9^. The CP is composed of a monolayer of epithelium cells secreting cerebrospinal fluid (CSF) and a monolayer of mesenchymal cells. Ins2 is expressed by the epithelium of the CP^9^. Therefore, insulin released from CP cells into the CSF may exert its effects on surrounding cells through the bulk flow of this liquid in the brain. However, at present, it is hard to distinguish between the exact contributions from CP insulin and β cell insulin.

Our data suggest that Ins2-Cre can be an alternative tool for β cell biology. The Cre/LoxP approach is widely utilized for investigating the function, development, and oncogenesis of β cells in mice. The most commonly used insulin-Cre driver lines have been Tg(Ins2-Cre)^25M*gn*^, Tg(Ins2-Cre)^23H*err*^, and Tg(Ins2-Cre/ERT)^1D*am*^, all of which are based on the use of a short rat *Ins2* promoter fragment (typically ~670 bp)^18^,^19^,^20^. However, a major shortcoming of these Cre lines is the leaky activity in the brain, which is most likely due to the absence of some essential regulatory elements. In addition, some lines exhibit abnormal glucose tolerance and insulin secretion due to the utilization of the human growth hormone minigene^32^,^33^,^34^, thereby becoming unsuitable to be used for the study of β cell function. Therefore, it is crucial to generate a new insulin-Cre driver line with high recombination efficiency and specificity. Currently, some promising *Ins1* promoter-based Cre driver lines have been generated with bacterial artificial chromosome (BAC) DNA or the knockin approach, which show straight or inducible Cre activity exclusively in β cells^35^,^36^,^37^,^38^. Nevertheless, given the high recombination efficiency in β cells and negative expression and activity in the hypothalamus, our Ins2-Cre knockin line should be a good alternative for studying β cell biology. One caveat exists when using this line to drive β cell-specific manipulation of a “floxed” gene of interest, which could also be manipulated in CP as well as in β cells. Although the role of CP in glucose, lipid, and energy homeostasis has not been validated, it should still be taken into account that the genetic manipulation of CP may contribute to the observed phenotypes.

In conclusion, this study reveals that the knockin of IRES-Cre into the *Ins2* locus allows high-fidelity detection of endogenous *Ins2* promoter activity without compromising insulin expression. In the brain, *Ins2* is mainly expressed in the CP but not in hypothalamus. Given its high efficiency of recombination in islet β cells, this Ins2-Cre line may represent a good alternative for exploring β cell biology.

## Materials and Methods

### Construction of the Ins2-Cre targeting vector

The knockin targeting vector was constructed using the λ phage Red recombination system. First, a retrieving vector, containing the third exon of the *Ins2* gene as a target site, was cloned by retrieving the genomic DNA fragment (~12 kb) of the mouse *Ins2* gene from the BAC clone 365C19 (Cell Biolabs, Inc., CA) via homologous recombination in the bacteria EL350. IRES-Cre-pA was constructed from the pFloxin-MCS2-IRES-MCS vector^39^ (Addgene #24642) and pGK-Cre-bpA (Addgene #11543), which harbors the bacteriophage P1 recombinase Cre with a SV40 large T antigen nuclear localization signal (NLS-Cre) and a bovine growth hormone polyadenylation site (114 bp)^23^. Then, a mini-targeting vector was cloned by inserting the IRES-Cre-bpA DNA sequence (vector #392) and the FRT-flanked Neo cassette between the stop codon and the 3′ UTR in the 3^rd^ exon. Last, the knockin targeting vector was generated by homologous recombination between the retrieving vector and the mini-targeting vector in EL350 and confirmed by DNA sequencing.

### Generation of Ins2-Cre mice

ES cells with 129Sv background were transfected with the targeting vector, and this was followed by positive and negative selections with G418 and ganciclovir. Homologous recombination at the Ins2 locus was confirmed by PCR analysis of the genomic DNA. The targeted ES cells carrying the Ins2-Cre/Neo allele were injected into C57BL/6 blastocysts to produce chimeric mice that transmitted the allele to the progeny. The neomycin resistance cassette was removed by breeding with Flp transgenic mice^24^, which was confirmed by PCR with the primers P3 and P6 flanking the neomycin resistance cassette. PCR primers for genotyping are available upon request. The PCR parameters used included 1 cycle of 95 °C for 5 min, 35 cycles of amplification (94 °C for 30 s, 57 °C for 30 s, and 72 °C for 90 s), and a single cycle of 72 °C for 5 min. Ten microliters of the PCR product was run on a 1% agarose gel for 30 min at 120 V. As a result, the heterozygotes with the Neo-deleted Ins2-Cre allele were used in all the experiments, and had the mixed genetic background of 129Sv and C57BL/6J. All animals were housed in a specific pathogen-free facility under controlled temperature and light and were fed with normal chow. All the experimental protocols were approved by the Animal Ethics Committee of Second Military Medical University, and the methods were carried out in accordance with the approved guidelines.

### Analysis of RNA

The hippocampus and hypothalamus were dissected from adult mice under a stereoscope. Islets were isolated by intraductal injection of collagenase P and handpicked under a stereoscope^40^. Total RNA was extracted from their TRIzol (Invitrogen) homogenates. Real-time RT-PCR was performed in a two-step reaction. First, the cDNA strand was synthesized with the Superscript III RT-PCR kit (Invitrogen), and then the second step was performed in a fluorescent temperature cycler (Mastercycler ep realplex, Eppendorf) with SYBR green and specific primers for each of the genes. Every plate included the 36B4 gene as internal control. Primer sequences are available on request. Results were analyzed with Student’s unpaired t-test.

### Histology and immunohistochemistry

Protocols for HE staining and the immunohistological analysis of hormone expression in pancreatic islets were previously described^40^. X-gal staining was performed as previously described^41^. Briefly, mice were perfused with 4% paraformaldehyde, and the tissues were further immersed in the fixative at 4 °C for 4 h prior to cryosectioning. Sections of 15-μm were stained at 37 °C for 24 h with a X-gal (5-bromo-4-chloro-3- indolyl-β-D-galactosidase) solution containing 1 mg/ml X-gal, 5 mM K_3_Fe(CN)_6_, 5 mM K_4_Fe(CN)_6_, and 2 mM MgCl_2_. The coronal forebrain sections and consecutive cryosections of the hypothalamus were prepared with thicknesses of 12 μm from the Ins2-Cre mice and visualized under a fluorescence microscope^42^.

### Metabolic analyses

A glucose tolerance test was performed by intraperitoneal injection of D-glucose (2 g/kg body weight) into overnight-fasted mice. For the *in vivo* insulin release assay, glucose (2 g/kg body weight) was intraperitoneally injected, and plasma insulin levels were detected at indicated time points by an ELISA kit (Mercodia Co.), as previously described^40^.

### Statistical analysis

All values, unless otherwise indicated, are expressed as the mean ± SEM. Statistical analyses were carried out using Student’s *t* test between two groups, and the null hypothesis was rejected at the 0.05 level.

## Additional Information

**How to cite this article**: Li, L. *et al*. Knockin of Cre gene at Ins2 locus reveals no Cre activity in mouse hypothalamic neurons. *Sci. Rep.* **6**, 20438; doi: 10.1038/srep20438 (2016).

## Supplementary Material

Supplementary Information
