# Supplementary material for: Knockin of Cre Gene at Ins2 Locus Reveals No Cre Activity in Mouse Hypothalamic Neurons
Source: Sci Rep. 2016 Feb 2;6:20438. doi: 10.1038/srep20438 (PMC4735843; doi:10.1038/srep20438)
Supplement: Supplementary Information [file srep20438-s1.pdf]

Supplementary Information

Knockin of Cre Gene at Ins2 Locus Reveals No Cre Activity in Mouse Hypothalamic Neurons

Lin Gao[1, 2*], Ling Li[1,2*], Kejia Wang[1,2*], Xianhua Ma[1,2], Xusheng Chang[1,2], Ye Zhang[1,2], Kai Yin[2,3], Zhimin Liu[2,4], Yuguang Shi[5], Zhifang Xie[1,2], Weiping J. Zhang[1,2]

[1]Department of Pathophysiology, [2]Center for Obesity & Diabetes Research and Innovation, Second Military Medical University, Shanghai 200433, China

[3]Department of General Surgery, Changhai Hospital, Shanghai, China

[4]Department of Endocrinology, Changzheng Hospital, Shanghai, China

[5]Barshop Institute for Longevity and Aging Studies, University of Texas Health Science Center at San Antonio, San Antonio, TX 78245, USA

Supplementary Figures 1-3.

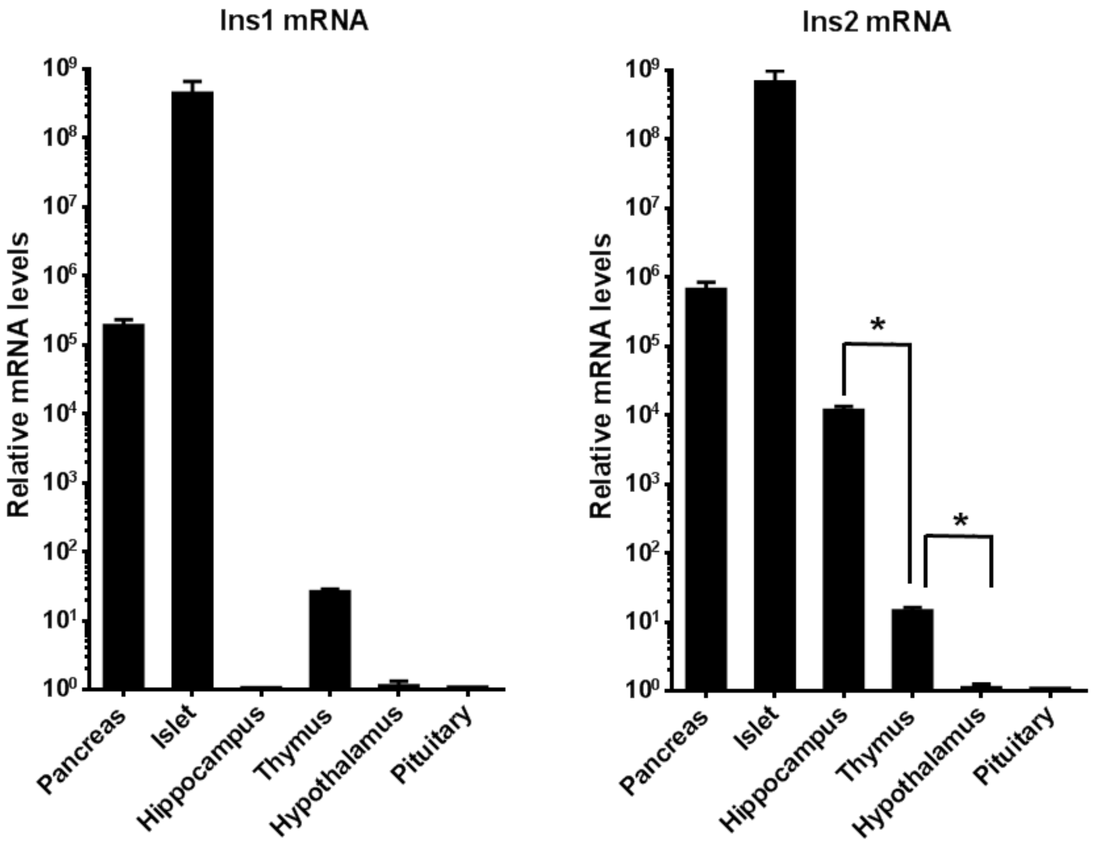

Supplementary Figure S1. Expression of insulin genes in the tissues from Ins2-Cre mice. mRNA levels of *Ins1* and *Ins2* were measured by quantitative RT-PCR in relevant tissues, and normalized by internal control 36B4. N=4. **P*<0.01

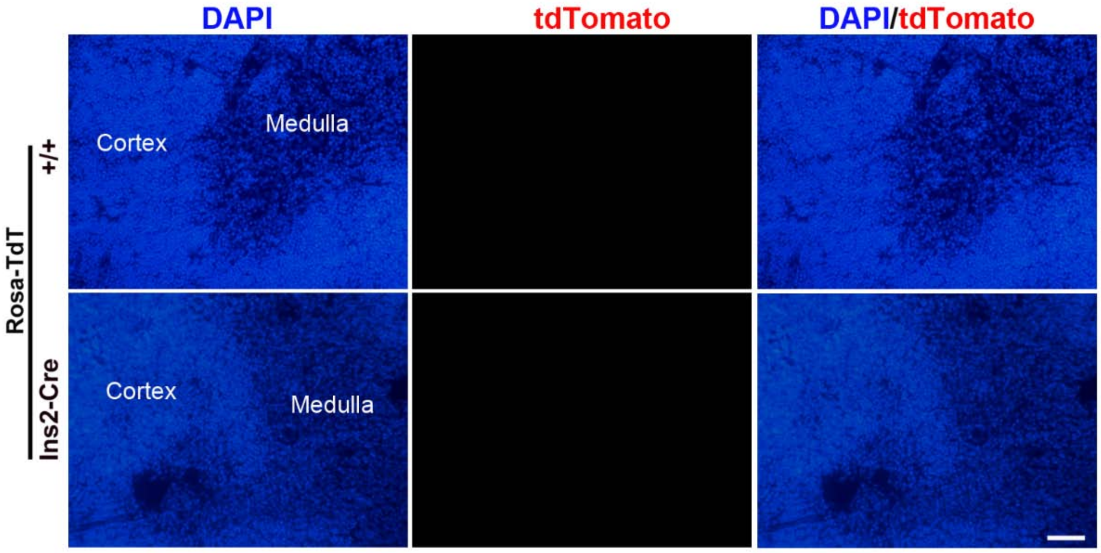

Supplementary Figure S2. Cre recombination activity is undetectable in the thymus from Ins2-Cre/Rosa-tdTomato mice. Representative thymus sections show that no significant tdTomato fluorescence is present in the thymus from adult Ins2-Cre;Rosa-tdTomato mice. Nuclei were stained blue with DAPI. Scale bar, 50 μm.

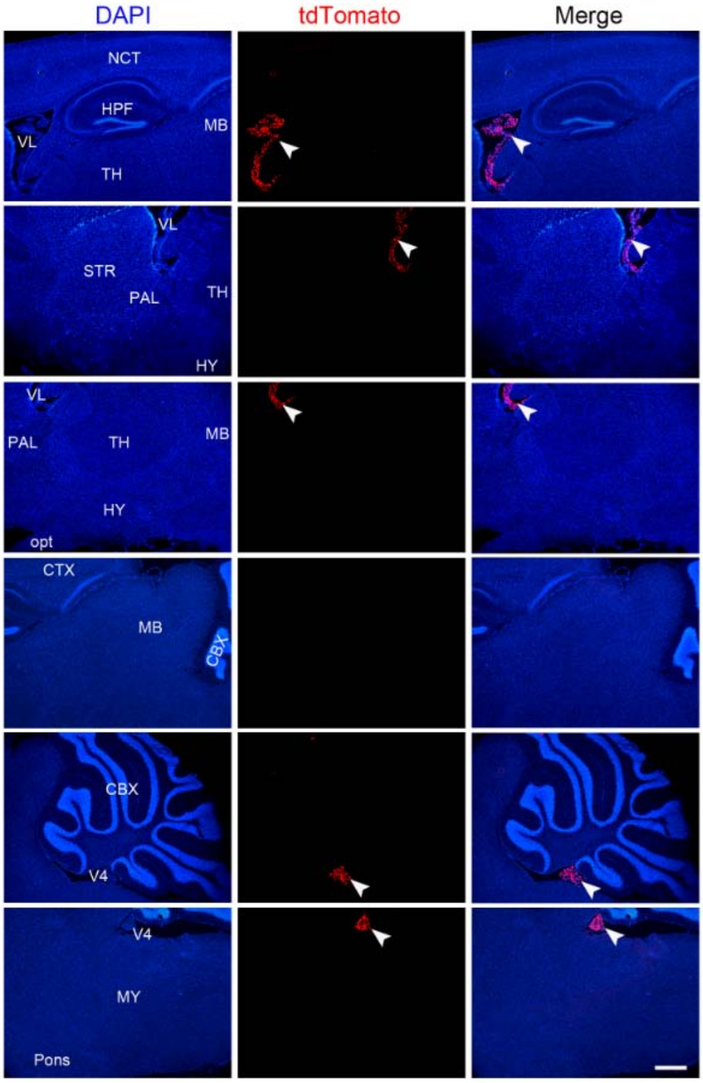

Supplementary Figure S3. Cre recombination activity is undetectable in the hypothalamus or other brain regions than choroid plexus in the Ins2-Cre/Rosa-tdTomato mice. Representative sagittal brain sections of Ins2-Cre;Rosa-tdTomato mice show that tdTomato is expressed by choroid plexus in the lateral (VL) and fourth ventricles （V4） (indicated by arrow heads) but undetectable in the neocortex (NCT), hippocampal formation (HPF), cerebral nuclei, thalamus (TH) , hypothalamus (HY), midbrain (MB), hindbrain, cerebellar cortex (CBX) andmedulla (MY). CTX, cerebral cortex; Opt, optic tract; PAL,pallidum;STR, striatum. Scale bar, 500 μm.
